# Intracisternal tuberculoma: a refractory type of tuberculoma indicating surgical intervention

**DOI:** 10.1186/s12883-017-0996-x

**Published:** 2018-01-18

**Authors:** Fanfan Chen, Lei Chen, Yongfu Cao, Yongjun Yi, Jingwen Zhuang, Wuhua Le, Wei Xie, Lanbo Tu, Peng Li, Yimin Fang, Ling Li, Yuqing Kou, Kaikai Fu, Hua He, Hongbin Ju

**Affiliations:** 1 Neurosurgery Department, Guangzhou First People’s Hospital, Guangzhou Medical University, 1# Panfu Road, Guangzhou, Guangdong 510180 China; 2 Neurosurgery Department, Shenzhen Second People’s Hospital, Shenzhen University, 3002# Sungang Road, Shenzhen, 518037 China; 3 Tuberculosis Department, Guangzhou Chest Hospital, 62# Hengzhi Gang Road, Guangzhou, Guangdong 510095 China; 4 Record Department, Guangzhou First People’s Hospital, Guangzhou Medical University, 1# Panfu Road, Guangzhou, Guangdong 510180 China; 5 Department of Navy Medicine, The Second Military Medical University, 800# Xiangyin Road, Shanghai, 200433 China; 6 Neurosurgery Department, Changzheng Hospital, The Second Military Medical University;State key Laboratory of Drug Research, Shanghai Institute of Material Medical, Chinese Academy of Sciences, 415# Fengyang Road, Shanghai, 200003 China; 7 Spinal Surgery Department, Guangzhou First People’s Hospital, Guangzhou Medical University, 1# Panfu Road, Guangzhou, Guangdong 510180 China

**Keywords:** Central nervous system, Paradoxical response, Tuberculosis, Spine, Tuberculoma

## Abstract

**Background:**

Central nervous system (CNS) tuberculoma is a rare disease with severe neurological deficits. This retrospective research is to review the data of patients diagnosed as CNS tuberculoma. Surgeries were performed in all patients. The clinical features especially the neurological image and the anatomical characters of the tuberculomas were concerned.

**Methods:**

Totally 11 patients diagnosed as CNS tuberculoma were admitted in Guangzhou First People’s Hospital (7cases) and Changzheng Hospital (4 cases) during 2006–2015. The data including preoperative condition, neurological imaging, and surgical findings was collected and analyzed.

**Results:**

The lesions of nine patients (9/11) were totally or subtotally excised and two (2/11) were partially excised. Neurological functions of all patients were improved after surgery without secondary infection. Lesions of nine (9/11) patients preoperatively progressed as a result of paradoxical reaction. Of the 9 patients demonstrated paradoxical progression, all lesions were partially or totally located at the cisterns or the subarachnoid space. Preoperative ATTs lasted 2 to 12 months and tuberculomas were not eliminated. The arachnoid was found thickened and tightly adhered to the lesions during surgeries. Of the 2 cases that paradoxical reaction were excluded, both patients (case 6, intramedullary tuberculoma; case 11, intradural extramedullary tuberculoma) were admitted at onset of the disease. ATTs were preoperatively given for 1 week as neurological deficits aggravated. The tuberculous lesions of CNS or other system showed no obvious change and paradoxical reaction could not be established in both cases.

**Conclusions:**

Exudates of tuberculosis is usually accumulated in the cisterns and frequently results in the paradoxical formation of tuberculoma. Intracisternal tuberculoma is closely related to paradoxical reaction and refractory to anti-tuberculosis therapy. Micro-surgical excision is safe and effective. Early surgical intervention may be considered in the diagnosis of intracisternal tuberculoma especially when paradoxical reaction participates in the development of tuberculoma.

## Background

Tuberculosis (TB) with central nervous system (CNS) involvement occurs in approximately 1% of all TB patients and causes the highest morbidity and mortality [1]. CNS tuberculosis has various forms. Tuberculous meningitis (TM) is the most frequent form of CNS TB and CNS tuberculoma is the type next to TM [2, 3]. Spinal intradural tuberculomas including intramedullary and intradural extramedullary tuberculoma are exceptionally rare and account for approximate 2%–5% of all CNS tuberculoma [4]. Furthermore, Intradural extramedullary tuberculoma of the spinal cord is the most unusual type [5].

Although CNS tuberculoma is an uncommon disease, it usually presents with severe neurological deficits including altered mental status, hydrocephalus, cranial nerve palsies, hemiparesis and seizures et al. [6]. Anti-tuberculosis treatments (ATT) combined with surgeries in the treatment of CNS tuberculoma have been reported occasionally [7, 8]. However, most of the articles mainly described the rarity of this disease and clinical situation including diagnosis, medications and outcomes of surgeries [9–12]. The locations, possible pathogenesis and the relation between them were not mentioned in those reported cases, except for the optochiasmatic tuberculoma [13].

Of the patients in this study, we were aware of the characteristics that the subarachnoid space including cerebral cisterns and spinal subarachnoid space were susceptible regions for the formation of tuberculomas following TM and/or tuberculous arachnoiditis. This situation was also noticed by other doctors [4, 14]. Paradoxical reaction, defined as an phenomenon of effective medical treatment demonstrating opposite effect in certain lesions [15, 16], is prone to occur at the cisterns and frequently results in the formation of intracisternal tuberculoma [17, 18]. In most cases, ATT is effective in treating the CNS tuberculoma [19]. Unfortunately, when paradoxical reaction participates in the etiology of tuberculoma, the effectiveness of ATT is usually limited and additional corticosteroids is indicated [20]. Nevertheless, long period of medical therapy for resolving the lesion results in limited improvement or even deterioration of the impaired neurological function, especially for the spinal paradoxical tuberculoma [2]. From the above information, intracisternal tuberculoma, paradoxical reaction and their intimate relation result in the severity and difficulty of medicine treatment. Accordingly, rapid elimination of the tuberculoma by surgical intervention should be considered.

## Methods

### Patients’ data

This retrospective research was approved by the ethics committee of Guangzhou First People’s Hospital and Changzheng Hospital. From 2006 to 2015, 11 patients diagnosed as CNS tuberculoma requiring surgical intervention were admitted in the neurosurgery department of Guangzhou First People’s Hospital, Guangdong, China and Changzheng Hospital, Shanghai. All patients underwent surgical excision of tuberculoma. Preoperative sputum smear was negative of tubercle bacillus of all patients. The diagnosis of tuberculosis of nine cases (pulmonary or CNS) was established in specialized hospital for tuberculosis. Two patients were diagnosed as spinal tuberculoma at onset of the disease and adequate ATT was administered for 1 week before sugeries. The age of patients ranged from 7 to 52 years old. Six patients were female and five were male. Of the nine patients with preoperative diagnosis of tuberculosis, effective ATT was applied. Effectiveness of ATT was evidenced by the improvement of the clinical symptom and radiological findings (including pulmonary or most part of the CNS lesions). However, new lesions or progression of one lesion was found in these nine patients in later period during the ATT. Paradoxical reactions were identified in these nine patients and additional corticosteroid was administered. Paradoxical reaction was not considered in the patient presenting intramedullary tuberculoma and the patient with intradural extramedullary tuberculoma of the fifth thoracic vertebra as both patients presented tuberculomas initially. For the two patients, surgeries were performed 1 week after ATT initiated as negative sputums for tubercle bacillus were confirmed. As shown in Table 1, the location of symptom related tuberculoma included the cerebral hemispheres (three cases), posterior fossa (three cases), and spinal (five cases, including one case of intramedullary tuberculoma and four cases of intradural extramedullary tuberculoma.).

**Table 1:** Clinical data of patients

Patients	Range of age (years)	Site /related cistern	Outcome of Initial tuberculosis after ATT	ATT time before CNS progression	Paradoxical reaction (Y/N)
1	50–55	Right Cerebello -pontine Angle	Relieved (cough, subcutaneous tuberculous nodule)	12 months	Y
2	15–20	Right Cisterna Magna	Improved (Fever, multiple organ tuberculosis)	3 months	Y
3	25–30	T3-T10, Intradural extra-medullary/intramedullary	Improved (Fever, pulmonary tuberculosis)	2 months	Y
4	15–20	Right Sylvian cistern	Improved (Fever, pulmonary tuberculosis)	10 months	Y
5	25–30	Left sylvian cistern	Improved (Fever) Progression (Headache)	6 months	Y
6	20–25	C5–6,intramedullary	Improved (Fever,pleural effussion), Progression (paraplegia)	1 week	N
7	10–15	Ambient cistern	Relieved (Pulmonary tuberculosis)	10 months	Y
8	40–45	T3–6 intradural extramedullary	Improved (Fever, pulmonary tuberculosis)	2 months	Y
9	15–20	T10–11 intradural extramedullary	Improved (Fever, pulmonary tuberculosis)	2 months	Y
10	5–10	T5 intradural extramedullary	Improved (Fever), Progression (paraplegia)	1 week	N
11	25–30	Left sylvian csitern	Improved (Fever) Progression (Headache)	6 months	Y

*T* thoracic, *C* cervical, *Y* yes, *N* no

### Preoperative preparations

All patients were given routine examinations of blood, blood electrolyte, liver function and kidney function. Electrocardiography and chest radiography were performed to exclude cardiac or pulmonary contraindications for surgeries. Sputum smears were performed repetitively assuring negative result of tubercle bacillus. MRI was acquired for a preoperative evaluation. ATT based on the regimen of specialized hospital for treatment of tuberculosis and was continued during the entire hospital stay of the patient.

### Surgical management

Two patients experienced two surgeries. One patient (patient 7) with tuberculoma locating at dorsal pons to cerebellum (cisterna ambiens) received external draininage of the tuberculoma at local hospital and relapsed after 2 months. A microsurgical excision was given by our team. Another patient (patient 3) with intradural extramedullary tuberculoma (co-existing with an intramedullary lesion situating at the ventral spinal cord without surgical intervention) underwent two surgeries for the intradural extramedullary tuberculoma. Other patients underwent operations once respectively. The symptom-inducing lesion was the surgical target in patient with multiple lesions. For example, patient 2 (Fig. 2c, d), the lesion locating at right cisterna magna (Fig. 2c, white arrow) was responsible for the hydrocephalus and hemiparesis. This lesion was the target of surgery. The lesion of left cerebellum was small and asymptomatic (Fig. 2c, d, red arrow). This lesion with other supratentorial lesions was resolved by the effective ATT. Precautions were taken to protect the surrounding normal brain tissue from surgical contamination. For the spinal tuberculomas, pedicle screw fixation surgeries were performed if necessary. All patients were followed until the present time. Follow-ups were performed by telephone or at outpatient department in all patients.

## Results

### Preoperative MRI findings

**Fig. 1:**
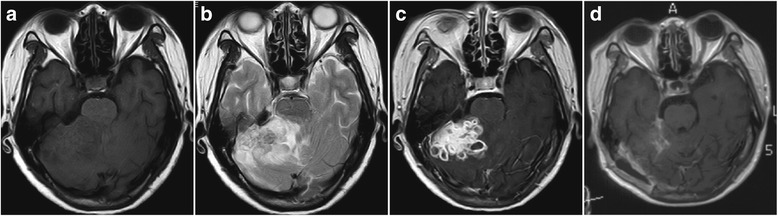
Typical MRI of intracisternal tuberculoma. **a** The lesions showed iso- and hypo-intense on T1WI. **b** Mixed signal intensity on T2WI. **c** Contrast-enhanced T1WI sequence showed multiple ring enhancing lesions. **d** The postoperative MRI of the patient

**Fig. 2:**
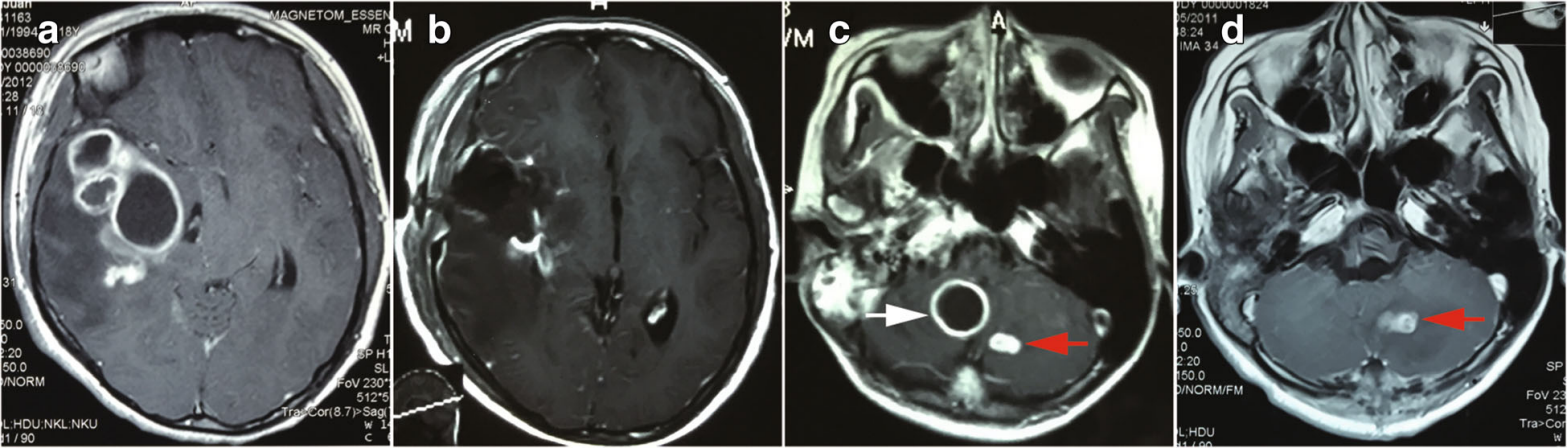
Pre and post-surgical image of intracisternal tuberculoma. **a** Preoperative enhancing MRI of a tuberculoma locating at right sylvian fissure. **b** Postoperative enhancing MRI of a tuberculoma locating at right sylvian fissure. **c** Preoperative enhancing MRI showed a tuberculoma locating at right cisterna magna (white arrow). The tuberculoma of left cerebellum (red arrow) was resolved by ATT. **d** Postoperative enhancing MRI showed the tuberculoma locating at right cisterna magna was excised. The tuberculoma of left cerebellum (red arrow) was resolved by ATT in later period

For CNS tuberculoma, MRI is the most important examination for preoperative preparation. The lesions showed hypo- to iso-intense on T1WI (Fig. 1a) and mixed signal intensity on T2WI (Fig. 1b). Contrast-enhanced T1WI sequence displayed isolated or conglomerated ring enhancing lesions accompanied with hypo-intense non-enhancing content inside in most situations (Fig. 1c, 2a, c).

**Fig. 3:**
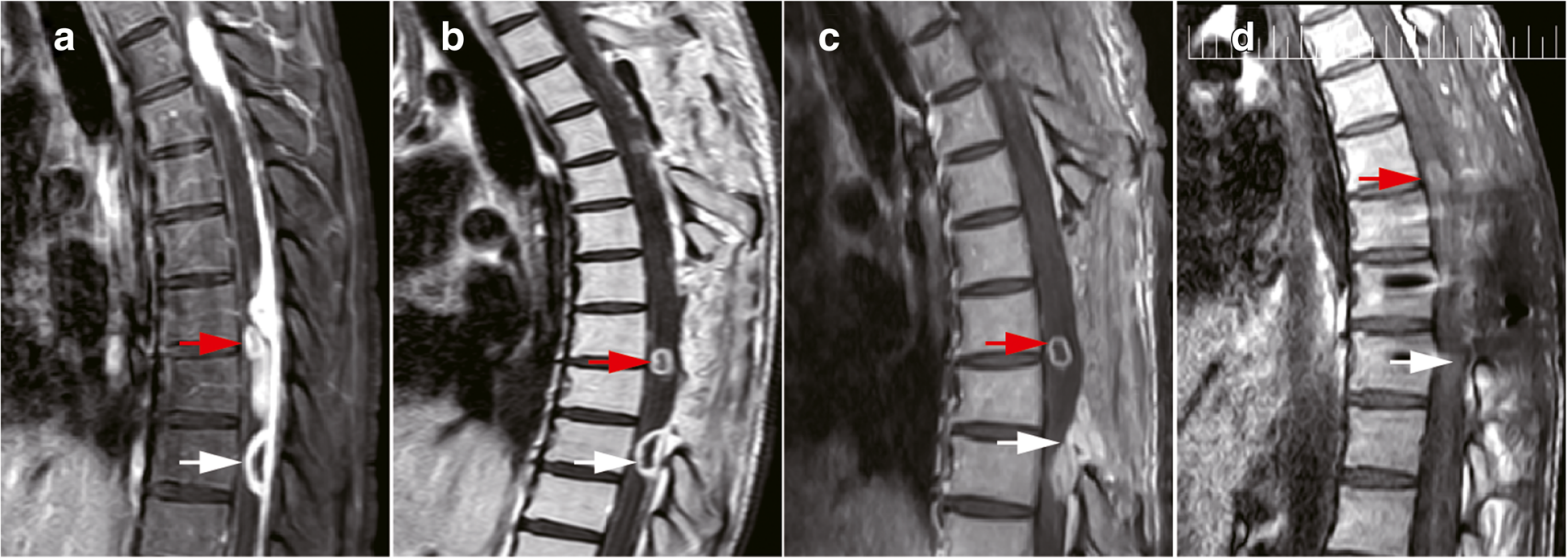
MRI of a patient with intradural extramedullary tuberculoma and intramedullary tuberculoma (two operations). **a** Preoperative MRI of the first operation of the patient manifesting intradural extramedullary tuberculoma from T3 to T9 (the enhancing lesion from T3 to T9) and intramedullary tuberculoma (red arrow). **b** Postoperative MRI of the first operation of the patient manifesting intradural extramedullary tuberculoma from T3 to T9 and intramedullary tuberculoma (red arrow). The intramedullary tuberculoma of T7 was left untouched while the intradural extramedullary lesion of T9 was missed (white arrow). **c** Preoperative MRI of the second operation of the patient. The intradural extramedullary tuberculoma of T9 was the target of operation (white arrow). **d** Postoperative MRI of the second operation of the patient. The intradural extramedullary tuberculoma of T9 was excised (white arrow)

This group of patients contained various locations of the CNS including temporal lobe, frontal lobe, cerebello-pontine angle, cerebella and intravertebral canal. It is noteworthy that except one intramedullary tuberculoma case, all other cases were associated with the subarachnoid space, including the sylvian fissure (Fig. 2a), cerebellopontine angle (Fig. 1c), ambient cistern (Fig. 5a), cerebellomedullary cistern (Fig. 2c) and the subarachnoid space of the spinal cord (Fig. 3a).

### Surgical findings

Patient 3 presented with coexisting intramedullary (Fig. 3a-d, red arrows) and intradural extramedullary lesions (Fig. 3,the continuous enhancing intradural extramedullary lesion from T3-T9). Preoperative evaluation identified that the lesions responsible for the symptoms were the multiple intradural extramedullary tuberculomas. Given that the intramedullary lesion was located at the ventral spinal cord, a direct excision of the intramedullary lesion might cause further spinal injury to the spinal function that was already seriously damaged, the intramedullary lesion was not resected. A laminectomy of T3-T4, T7-T9 or the excision of the corresponding extramedullary lesions was performed (Fig. 3b). However, the lesion of T9 was missed(Fig. 3b, white arrow). After 2 months of ATT, the following situations were displayed: extramedullary lesion of T9 enlarged (Fig. 3c, white arrow); the residual lesion of T5-T6 disappeared; the intramedullary lesion showed no change (Fig. 3a-c). A second time surgery was performed to remove the progressing extramedullary lesion of T9. At the same time, a pedicle screw fixation was performed to maintain the stability of the spinal column (Fig. 3d).

**Fig. 4:**
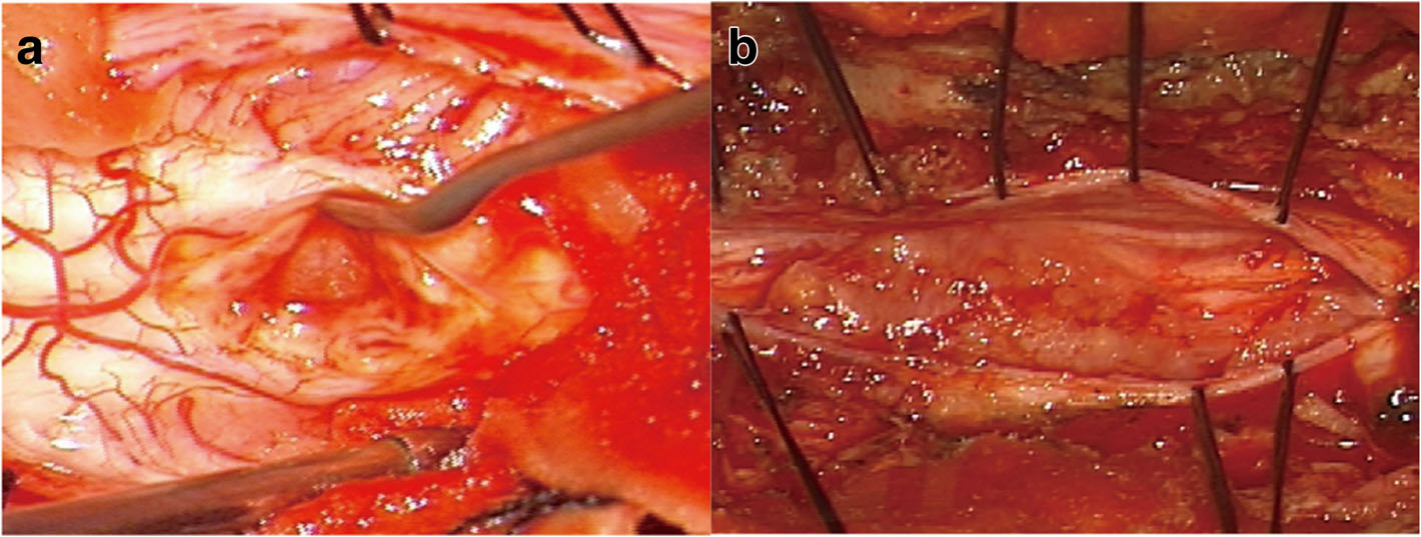
Tuberculomas at different regions displayed different intraoperative situation. **a** Intramedullary tuberculoma displayed clear boundary and was easily separated. **b** Extramedullary tuberculoma displayed diffusive lesion with arachnoiditis which was tightly adhered to the spine and difficult for separation

**Fig. 5:**
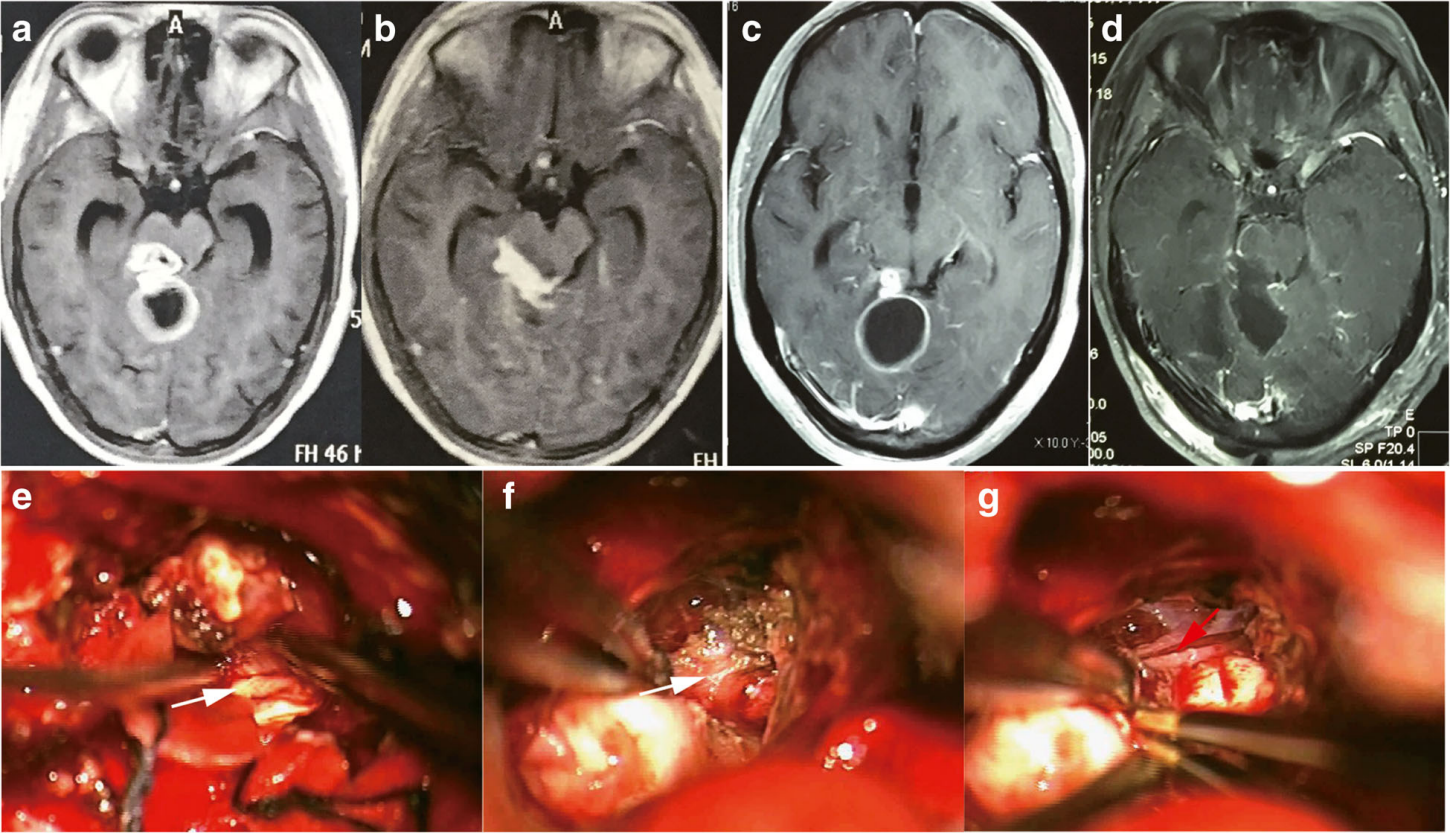
The MRI and intraoperative situation of a tuberculoma associated with ambient cistern. **a** Preoperative MRI of the lesion. **b** Drainage surgery was performed for the first time operation at local hospital with residue mainly at the ambient cistern. **c** The tuberculoma was relapsed with effective ATT and corticosteroids. **d** A total resection of the lesion was done. **e** Intraoperative image showed the clear boundary of the part of tuberculoma situating at cerebellum parenchyma (white arrow). **f** The intracisternal part of the same tuberculoma demonstrated tightly adhesion to the thickening arachnoid which was relatively difficult for separation (white arrow). **g** Exposure of a vessel coursed in the subarachnoid space (red arrow)

The intramedullary tuberculoma was revealed to be well-defined lesions (Fig. 4a) which is totally different for the intradural extramedullary tuberculoma. As for the latter, the abscess extended on the surface of spinal cord (Fig. 4b). The irregular lesion adhere to surrounding tissues intimately. Similar situation was oberserved in the cerebral tuberculoma. Even for the same tuberculoma, the interface between the tuberculoma and parenchyma was relatively loose and easy to be separated (Fig. 5e, white arrow displayed the interface of the tuberculoma and the parenchyma of brain) while the adhesion of tuberculoma in the subarachnoid space was tight (Fig. 5f, white arrow displayed the adhesion of tuberculoma and arachnoid). This kind of close relation between subarachnoid space and the cisternal part of tuberculoma was due to the thickening arachnoid, the hyperplastic fibrous tissue closely surrounding the lesion. Coagulation and sharp dissection is necessary for separation. Furthermore, manipulation in the cisternal part of tuberculoma should be more careful as vital vessel may course in the cistern. Decisions of cutting or separation and reservation the vessel should be done based on clear identification (Fig. 5g, red arrow indicates the vessel after separation of arachnoid).

None of the patients experienced secondary infection following the surgical procedures. All of the patients were followed from the date of surgery until the present time. The outcomes of all patients were evaluated with Karnofsky Performance Scale in different neurological recovery periods. The recovery times of patients in this study were different. For the most severe case as case 3, 40 months had passed before the patient finally carried out daily activities (KPS was 90). For the other patient, it took about 2–6 months for recovery (KPS were 90–100).

### Pathology

**Fig. 6:**
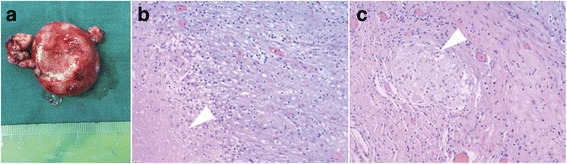
The specimen of an intracisternal tuberculoma and the pathological examination. **a** The specimen of an intracisternal tuberculoma (patient 7). **b** Typical image showed the caseous necrosis of the specimen (arrow), epithelial cell and lymphocyte. **c** Typical image showed the tuberculous granuloma (arrow), epithelial cell and lymphocyte

All specimens were pathologically analyzed. The pathological characteristics including granulomas, necrosis, caseation, cell types (lymphocyte, Langerhans-type giant cell), and the result of acid-fast bacillus staining were recorded. Not all the pathological features were demonstrated in one case. Typical tuberculomas showed a granulomatous reaction consisting of epithelioid cells and giant cells mixed with mononuclear inflammatory cells (predominantly lymphocytes) that form a granuloma (Fig. 6b, c) [6, 21].

## Discussion

CNS tuberculoma is a devastating disease with high morbidity and mortality. Although the outcome of the patients in this study was encouraging, the periods of preoperative ATT (for the paradoxical tuberculoma) were 2 to 12 months (average 5.9 ± 4.0 months). Neurological functions in most cases were aggravated during the ATT treatment as a result of a paradoxical reaction which limited the effect of ATT. Surgical excision of the lesion was a turning point for this group of patients. To our opinion, early surgical excision is an appropriate procedure when a paradoxical lesion formed. This is particularly critical for spinal tuberculoma [1, 22].

Although the clinical situations of the patients were different, for example the sites of the lesions and the response to treatment, we noticed that the tuberculoma of nine (9/11) patients who were considered paradoxical tuberculoma were correlated with the subarachnoid space. Furthermore, it was intriguing that the relapsed lesions of the two patients who experienced local recurrence were located at the dorsal ambient cistern or spinal subarachnoid space, respectively. We identified the thickening arachnoid adhered to the lesion during the surgery as described in previous literature [8]. The subarachnoid space involvement accompanied with the thickening arachnoid seems to be a potential risk factor for the occurrence and recurrence of tuberculoma. Meanwhile, frequent occurence of paradoxical reaction in the subarachnoid space furtherly proved the refractory nature of intracisternal tuberculoma.

### Tuberculoma is frequently developed in subarachnoid space as a result of paradoxical reaction

The classical model of TM pathology suggests two steps of the pathological process of tuberculosis meningitis. Firstly, the mycobacteria begins to grow at the parenchyma or meninges of the brain or spinal cord, and then develops and matures into a tuberculous abscess. Secondly, the abscess breaks up and releases its contents into the subarachnoid space which causes TM [23]. TM may also be the result of the direct hematogenous spread of pulmonary tuberculosis [1]. Based on above knowledge, tuberculoma and TM may be considered as the initial manifestations of the CNS tuberculous pathology. More importantly, the subarachnoid space is a pathological and anatomical region where mycobacteria and its products frequently exist. The exudates of TM and the arachnoiditis usually accumulate at the subarachnoid space including the interpeduncular cisterns, optochiasmatic cistern, the ambient and the suprasellar cisterns, the sylvian fissures as well as the subarachnoid space of spine [17]. Spinal subarachnoid space is anatomically similar to the cerebral cisterns. This explained the facts that intradural extramedullary tuberculoma was closely related to the arachnoiditis as well [14]. Pathological progression following cisternal exudates includes arachnoiditis and tuberculoma [4, 11, 24]. This kind of cisternal tuberculoma displayed its own features other than the parenchymal tuberculoma. The gelatinous exudate is constrained by the hyperplastic thickened arachnoid whereas the exudate still could spread in the cistern. This illustrates multiple lobular tuberculoma is common in the images of published articles and in our cases which is different from the parenchymal tuberculoma (Fig. 1c, 2a) [8]. The adhesion of arachnoid led to the formation of the inter-septum of the tuberculoma. More importantly, the exudates in the cistern was considered a form of the paradoxical response [17]. Accordingly, tuberculoma secondary to the cisternal exudate was a product of paradoxical reaction too. It was reported the optochiasmatic region had a high propensity for accumulating a quantity of exudates, which was a frequent type of paradoxical reaction [25].

### Intracisternal tuberculoma is refractory to ATT and an indication for surgical intervention

Based on the above information, because the cisternal tuberculoma shows a high rate of paradoxical development, the effectiveness of ATT is limited [23]. The immunological reaction based paradoxical reaction results in the progression of the lesion which calls for additional corticosteroids but no needs of change of ATT regimen [20]. Thus, prolonged therapeutic time is inevitable. At the same time, the paradoxical progression of the intracisternal lesion furtherly damages the neurological function which indicates surgery. Natarajan et al. believed that paradoxical intraduaral extramedullary tuberculoma was an indication for surgery [7]. According to our findings, the relatively enclosed space of a tuberculoma-occupying cistern which impedes the flow of CSF may result in a low concentration of medicine in the area. In the two relapsed cases reported in our study, the lesions were both situated in the subarachnoid space. As for the case of the tuberculoma locating at the ambient cistern to the cerebellum, even the drainage surgery had a limited effect and did not change the microenvironment of the intracisternal tuberculoma (case 7). Finally, the safety of microsurgical excision had been proven as no secondary infection or dissemination was observed in literatures or our cases [9, 26]. All these factors ensure us that surgical resection should be early considered for the intracisternal tuberculoma.

### Intracisternal tuberculoma is accompanied with serious neurological deficits requiring early resolving of the lesions

As mentioned above, Intracisternal tuberculoma is frequently progressed from paradoxical reaction and fast elimination of the lesions was usually difficult. It is worse that the exudates, arachnoiditis and/or tuberculoma developing in the cistern and adjacent parenchyma furtherly damage the neurological functions [23]. Tuberculoma in the cistern blocks the circulation of CSF resulting in hydrocephalus. The mass effect and surrounding edema may lead to the occurrence of hernia or the compression of vital structures of the CNS in certain areas. The exudates and tuberculoma can affect the blood vessels arranged in the area causing vasculitis and subsequently stroke [27]. In a univariate analysis, sylvian fissure exudates were predictors of stroke [28]. The critical site of the intracisternal tuberculoma also requires more attention especially surgical consideration.

## Conclusions

Intracisternal and intradural extramedullary tuberculoma are specific forms of tuberculoma requiring particular attention. Exudates accumulation and arachnoiditis are the pathological changes occurring in these areas. Meanwhile, paradoxical reaction frequently participates in the formation of tuberculoma in these regions. This kind of intracisternal tuberculoma is refractory to ATT and corticosteroids which may take a long period to get the lesion resolved and potentially induce severe neurological injury. Surgical excision is an inevitable and necessary procedure for intracisternal tuberculoma for improving neurological function.

## Abbreviations

ATT: Anti-tuberculosis treatment
CNS: Central nervous system
CSF: Cerebral spinal fluid
MRI: Magnetic resonance imaging
TB: Tuberculosis
TM: Tuberculous meningitis
